# Supplementary material for: Machine Learning for Neural Decoding
Source: eNeuro. 2020 Aug 27;7(4):ENEURO.0506-19.2020. doi: 10.1523/ENEURO.0506-19.2020 (PMC7470933; doi:10.1523/ENEURO.0506-19.2020)
Supplement: Figure 4-3 — Additional decoder details, including equations and hyperparameters. These details are for the decoder implementations that we use in our demonstrations and have in our code package. Download Figure 4-3, PDF file. [file enu-eN-MNT-0506-19-s06.pdf]

Method	Equations, details, and hyperparameters
General information	For all the methods below, we describe *our particular uses*, which are available in our code package and are used in the decoding demonstrations we show in *Results*.<br><br>Let $\boldsymbol{X}$ be the input matrix of covariates and let $\boldsymbol{Y}$ be the output that is being predicted. $\boldsymbol{Y}$ is either a vector or matrix depending on whether the outputs (e.g. x and y components of velocity) are predicted simultaneously.<br><br>Also, note that for our demonstrations, for all methods below, the noise of the output is assumed to be normally distributed.
Wiener Filter	Decoder predictions have the form $\boldsymbol{Y} = \boldsymbol{\theta X}$, where $\boldsymbol{\theta}$ is fit according to maximum likelihood estimation.<br>*Hyperparameters:* None
Wiener Cascade	Decoder predictions have the form $\boldsymbol{Y} = \beta_0 + \beta_1 \boldsymbol{Z}^1 + \cdots + \beta_n \boldsymbol{Z}^n$, where $\boldsymbol{Z} = \boldsymbol{\theta X}$. The parameters $\boldsymbol{\theta}$ and the β's are fit iteratively according to maximum likelihood estimation.<br>*Hyperparameters:* Degree of the polynomial used for the nonlinearity, *n.*
SVR	Tutorials have previously been written on the details of support vector regression and how they are optimized (Smola and Schölkopf, 2004). Here, we provide a very short mathematical summary. In nonlinear SVR:<br><br>$$\boldsymbol{Y} = \sum_{i=1}^{l} (\alpha_i - \alpha_i^*) k(\boldsymbol{X_i}, \boldsymbol{X}) + b$$<br><br>where *i* represents the data point. *k* is the kernel function, which allows for the nonlinear transformation. We used a radial basis function kernel:<br><br>$$k(\boldsymbol{x}', \boldsymbol{x}) = \exp\left(-\frac{\lVert \boldsymbol{x}' - \boldsymbol{x} \rVert^2}{\boldsymbol{\sigma}^2}\right)$$<br><br>The parameters α_i and α_i^* are constrained to be in the range $[0, C]$ in the optimization problem, where C is a hyperparameter that can be understood as being inversely related to regularization strength.<br>*Hyperparameters:* Penalty of the error term (C), maximum number of iterations
XGBoost	In gradient-boosted tree methods (which XGBoost is an implementation of), a mapping is learned using a collection of regression trees. Training an individual regression tree is accomplished by sequentially splitting the input space, and assigning an output value to each final partition of the input space (see (Breiman, 2017) for further description). Let f_i be the input/output mapping of regression tree *i.* The final decoder, which combines across N regression trees, will have the form:<br><br>$$\boldsymbol{Y} = \sum_{k=1}^{N} f_k(\boldsymbol{X})$$

	To train the XGBoost decoder, regression trees are trained sequentially. The first tree is trained to minimize the loss function which here is $\|\boldsymbol{Y} - f_1\boldsymbol{X}\|_2^2 + \Omega(f_1)$, where Ω is a regularization function. The second tree is then trained to predict the residual of the first tree's predictions. That is, we optimize f_2 to minimize $\|\boldsymbol{Y} - f_1\boldsymbol{X} - f_2\boldsymbol{X}\|_2^2 + \Omega(f_2)$. Successive trees continue to be optimized to predict the remaining residual. See https://xgboost.readthedocs.io/en/latest/tutorials/model.html for a more in-depth tutorial description.<br>*Hyperparameters:* Maximum depth of each regression tree, number of trees, learning rate
Feedforward Neural Net	We created a fully connected (dense) feedforward neural network with 2 hidden layers and rectified linear unit ("ReLU") (Glorot et al., 2011) activations after each hidden layer. We required the number of hidden units in each layer to be the same. This can be written as:<br>$\boldsymbol{H_1} = \text{ReLU}(\boldsymbol{W_1X} + \boldsymbol{b_1})$<br>$\boldsymbol{H_2} = \text{ReLU}(\boldsymbol{W_2X} + \boldsymbol{b_2})$<br>$\boldsymbol{Y} = \boldsymbol{W_3H_2} + \boldsymbol{b_3}$<br>We used the Adam algorithm (Kingma and Ba, 2014) as the optimization routine to solve for all $\boldsymbol{W}$'s and $\boldsymbol{b}$'s. We used the Keras library (Chollet, 2015) for all neural network implementations. This neural network, and all neural networks below had two output units. That is, the same network predicted the x and y components (of position or velocity) together, rather than separately.<br>*Hyperparameters:* Number of units (dimensionality of $\boldsymbol{H_1}$ and $\boldsymbol{H_2}$), amount of dropout during training, number of training epochs
Simple RNN	Decoder predictions have the form*:*<br>$\boldsymbol{H_t} = \text{ReLU}(\boldsymbol{UX_t} + \boldsymbol{WH_{t-1}} + \boldsymbol{b})$<br>$\boldsymbol{Y_t} = \boldsymbol{VH_t} + \boldsymbol{c}$<br>Note that we used the ReLU nonlinearity as opposed to the "tanh" nonlinearity, which is more common in RNNs, as this improved performance. We used RMSprop (Tieleman and Hinton, 2012) as the optimization routine.<br>*Hyperparameters:* Number of units (dimensionality of $\boldsymbol{H}$), amount of dropout during training, number of training epochs
GRUs	Here, we mix notation from (Goodfellow et al., 2016) and (Olah, 2015), which provide excellent descriptions of the method. The GRU has two gates, which control the transmission of information through the network. The "update" and "reset" gates are parameterized as follows:<br>Update gate: $\boldsymbol{z_t} = \sigma(\boldsymbol{U_zX_t} + \boldsymbol{W_zH_{t-1}} + \boldsymbol{b_z})$<br>Reset gate: $\boldsymbol{r_t} = \sigma(\boldsymbol{U_rX_t} + \boldsymbol{W_rH_{t-1}} + \boldsymbol{b_r})$<br>A temporary hidden state is calculated as:

	$\widetilde{\boldsymbol{h}}_{\boldsymbol{t}} = \tanh(\boldsymbol{U_h X_t} + \boldsymbol{r_t} * \boldsymbol{W_h H_{t-1}} + \boldsymbol{b_c})$<br><br>In this calculation, the reset gate controls what information from the previous hidden state is in this proposed new hidden state. Note that the $*$ symbol denotes an elementwise (by neural network unit) multiplication. To calculate the hidden state, we use the update gate to control what information flows from the previous hidden state versus what is updated from the temporary state:<br><br>$\boldsymbol{h_t} = (\boldsymbol{1} - \boldsymbol{z_t}) * \boldsymbol{h_{t-1}} + \boldsymbol{z_t} * \widetilde{\boldsymbol{h}}_{\boldsymbol{t}}$<br><br>Finally, as in the standard RNN:<br>$\boldsymbol{Y_t} = \boldsymbol{VH_t} + \boldsymbol{c}$<br><br>We used RMSprop (Tieleman and Hinton, 2012) as the optimization routine.<br><br>*Hyperparameters:* Number of units (dimensionality of $\boldsymbol{H}$), amount of dropout during training, number of training epochs.
LSTM	As above, we mix notation from (Goodfellow et al., 2016) and (Olah, 2015), which provide excellent descriptions of the method. The LSTM has three gates, which control the transmission of information through the network. The "forget", "input", and "output" gates are parameterized as follows:<br><br>$\boldsymbol{f_t} = \sigma(\boldsymbol{U_f X_t} + \boldsymbol{W_f H_{t-1}} + \boldsymbol{b_f})$<br>$\boldsymbol{i_t} = \sigma(\boldsymbol{U_i X_t} + \boldsymbol{W_i H_{t-1}} + \boldsymbol{b_i})$<br>$\boldsymbol{o_t} = \sigma(\boldsymbol{U_o X_t} + \boldsymbol{W_o H_{t-1}} + \boldsymbol{b_o})$<br><br>The LSTM has a cell state, $\boldsymbol{C_t}$, that carries information across time. We can calculate $\boldsymbol{C_t}$, as:<br><br>$\widetilde{\boldsymbol{C}}_{\boldsymbol{t}} = \tanh(\boldsymbol{U_c X_t} + \boldsymbol{W_c H_{t-1}} + \boldsymbol{b_c})$<br>$\boldsymbol{C_t} = \boldsymbol{f_t} * \boldsymbol{C_{t-1}} + \boldsymbol{i_t} * \widetilde{\boldsymbol{C}}_{\boldsymbol{t}}$<br><br>where $\widetilde{\boldsymbol{C}}_{\boldsymbol{t}}$ is a temporary cell state, that becomes part of the cell state depending on the input gate, $\boldsymbol{i_t}$. Additionally, the previous time point's cell state is carried through depending on the forget gate, $\boldsymbol{f_t}$. Note that the $*$ symbol denotes an elementwise (by neural network unit) multiplication.<br><br>The hidden state of the network is an output-gated version of the cell state:<br>$\boldsymbol{H_t} = \boldsymbol{o_t} * \tanh(\boldsymbol{C_t})$<br><br>Finally, as in the standard RNN:<br>$\boldsymbol{Y_t} = \boldsymbol{VH_t} + \boldsymbol{c}$<br><br>We used RMSprop (Tieleman and Hinton, 2012) as the optimization routine.<br><br>*Hyperparameters:* Number of units (dimensionality of $\boldsymbol{H}$), amount of

	dropout during training, number of training epochs
Kalman Filter	In the standard Kalman Filter, <br>$$\boldsymbol{y}_t = \boldsymbol{A}\boldsymbol{y}_{t-1} + \boldsymbol{w}$$ <br>where $\boldsymbol{y}_t$ and $\boldsymbol{y}_{t-1}$ are 6 x 1 vectors reflecting kinematic variables (not just the velocity or position being predicting), and $\boldsymbol{w}$ is sampled from a normal distribution, $N(0,\boldsymbol{W})$, with mean 0 and covariance $\boldsymbol{W}$. <br>$$\boldsymbol{x}_{t*} = \boldsymbol{H}\boldsymbol{y}_t + \boldsymbol{q}$$ <br>where $\boldsymbol{x}_{t*}$ is the neural activity at time t^*, and $\boldsymbol{q}$ is sampled from a normal distribution, $N(0,\boldsymbol{Q})$. Note that we allowed a lag between the neural data and predicted kinematics, which is why we use t^* for the time of the neural activity. <br>During training, $\boldsymbol{A}$, $\boldsymbol{H}$, $\boldsymbol{W}$, and $\boldsymbol{Q}$ are empirically fit on the training set using maximum likelihood estimation. When making predictions, to update the estimated hidden state at a given time point, the updates derived from the current measurement and the previous hidden states are combined. During this combination, the noise matrices give a higher weight to the less uncertain information. See (Wu et al., 2003) or our code for the update equations (note that $\boldsymbol{x}$ and $\mathbf{y}$ have different notation in (Wu et al., 2003)). <br>We had one hyperparameter which differed from the standard implementation (Wu et al., 2003). We divided the noise matrix associated with the transition in kinematic states, $\boldsymbol{W}$**,** by the hyperparameter scalar C, which allowed weighting the neural evidence and kinematic transitions differently. The rationale for this addition is that neurons have temporal correlations, which make it desirable to have a parameter that allows changing the weight of the new neural evidence. The introduction of this parameter made a big difference for the hippocampus dataset (Extended Data Fig. 4 - 1). <br>*Hyperparameters:* C and the lag between the neural data and predicted kinematics.
Naïve Bayes	We used a Naïve Bayes decoder similar to the one implemented in (Zhang et al., 1998). We first fit an encoding model (tuning curve) using the output variables. Let $f_i(\boldsymbol{s})$ be the value of the tuning curve (the expected number of spikes) for neuron i at the output variables $\boldsymbol{s}$. Note that $\boldsymbol{s}$ is a vector containing the two output variables we are predicting (x and y positions/velocities). We assume the number of recorded spikes in the given bin, r_i, is generated from the tuning curve with Poisson statistics: <br>$$P(r_i\|\boldsymbol{s}) = \frac{\exp\left[-f_i(\boldsymbol{s})\right] f_i(\boldsymbol{s})^{r_i}}{r_i!}$$ <br>We also assume that all the neurons' spike counts are conditionally independent given the output variables, so that:

$$P(\boldsymbol{r}|s) \propto \prod_i P(r_i|s)$$

where $\boldsymbol{r}$ is a vector with the spike counts of all neurons. Bayes' rule can then be used to determine the likelihood of the output variables given the spike counts of all neurons:

$$P(\boldsymbol{s}|\boldsymbol{r}) \propto P(\boldsymbol{r}|\boldsymbol{s})P(\boldsymbol{s})$$

where $P(\boldsymbol{s})$ is the probability distribution of the output variables. To help with temporal continuity of decoding, we want our probabilistic model to include how the output variables at one time step depend on the output variables at the previous time step: $P(\boldsymbol{s}_t|\boldsymbol{s}_{t-1})$. Thus, we can more generally write, using Bayes' rule as before:

$$P(\boldsymbol{s}_t|\boldsymbol{r}_{t*}, \boldsymbol{s}_{t-1}) \propto P(\boldsymbol{r}_{t*}|\boldsymbol{s}_t)P(\boldsymbol{s}_{t-1}|\boldsymbol{s}_t)P(\boldsymbol{s}_t)$$

Note that we use $\boldsymbol{r}_{t*}$ rather than $\boldsymbol{r}_t$ because we use neural responses from multiple time bins to predict the current output variables. The above formula assumes that $\boldsymbol{r}_{t*}$ and $\boldsymbol{s}_{t-1}$ are independent, conditioned on $\boldsymbol{s}_t$. The final decoded stimulus in a time bin is: $\text{argmax}_{\boldsymbol{s}_t} P(\boldsymbol{s}_t|\boldsymbol{r}_{t*}, \boldsymbol{s}_{t-1})$.

$P(\boldsymbol{s}_{t-1}|\boldsymbol{s}_t)$ was determined as follows. Let $\Delta\boldsymbol{s}$ be the Euclidean distance in $\boldsymbol{s}$ from one time step to the next. We fit $P(\Delta\boldsymbol{s})$ as a Gaussian using data from the training set. $P(\boldsymbol{s}_{t-1}|\boldsymbol{s}_t)$ was approximated as $P(\Delta\boldsymbol{s}_t)$. That is, the probability of going from one output state to another was only based on the distance between the output states, not the output state itself.

Additionally, including $P(\boldsymbol{s})$ based on the distribution of output variables in the training set did not improve performance on the validation set. This could be because the probability distribution differed between the training and validation/testing sets, or because the distribution of output variables was approximately uniform in our tasks. Thus, we simply used a uniform prior.

In our calculations, we discretize $\boldsymbol{s}$ into a 100 x 100 grid going from the minimum to maximum of the output variables. When increasing the decoding resolution of the output variables, we did not see a meaningful change in decoding accuracy.

Our tuning curves had the format of a Poisson generalized quadratic model (Park et al., 2013), which improved the performance over generalized linear models on validation datasets.

On the hippocampus dataset, we used the total number of spikes over the same time interval as we used for the other decoders (4 bins before, the concurrent bin, and 5 bins after). Note that using a single time bin of spikes led to very poor performance. On the motor cortex and somatosensory

	cortex datasets, the naïve Bayes decoder gave very poor performance regardless of the bins used. We ultimately used bins that gave the best performance on a validation set: 2 bins before and the concurrent bin for the motor cortex dataset; 1 bin before, the concurrent bin, and 1 bin after for the somatosensory cortex dataset.<br><br>*Hyperparameters:* None

Figure 4-3: Additional decoder details, including equations and hyperparameters
These details are for the decoder implementations that we use in our demonstrations and have in our code package.

References

Breiman L (2017) Classification and regression trees: Routledge.

Chollet F (2015) Keras.

Glorot X, Bordes A, Bengio Y (2011) Deep sparse rectifier neural networks. In: Proceedings of the Fourteenth International Conference on Artificial Intelligence and Statistics, pp 315-323.

Goodfellow I, Bengio Y, Courville A (2016) Deep learning: MIT press.

Kingma D, Ba J (2014) Adam: A method for stochastic optimization. arXiv preprint arXiv:14126980.

Olah C (2015) Understanding LSTM Networks. Available from: https://colah.github.io/posts/2015-08-Understanding-LSTMs/.

Park IM, Archer EW, Priebe N, Pillow JW (2013) Spectral methods for neural characterization using generalized quadratic models. In: Advances in neural information processing systems, pp 2454-2462.

Smola AJ, Schölkopf B (2004) A tutorial on support vector regression. Statistics and computing 14:199-222.

Tieleman T, Hinton G (2012) Lecture 6.5-RmsProp: Divide the gradient by a running average of its recent magnitude. COURSERA: Neural Networks for Machine Learning.

Wu W, Black MJ, Gao Y, Serruya M, Shaikhouni A, Donoghue J, Bienenstock E (2003) Neural decoding of cursor motion using a Kalman filter. In: Advances in neural information processing systems, pp 133-140.

Zhang K, Ginzburg I, McNaughton BL, Sejnowski TJ (1998) Interpreting neuronal population activity by reconstruction: unified framework with application to hippocampal place cells. J Neurophysiol 79:1017-1044.
